# Treatment-refractory immune checkpoint inhibitor-induced hemophagocytic lymphohistiocytosis in the setting of adjuvant pembrolizumab for resected stage IIC melanoma: a case report

**DOI:** 10.1186/s13256-025-05302-2

**Published:** 2025-10-23

**Authors:** Kar Ven Cavan Chow, Kimberley Mui, Stewart Hunt, Chun Loo Gan

**Affiliations:** 1 Department of Medical Oncology, Royal Brisbane and Women’s Hospital, Butterfield St, Herston, QLD 4006 Australia; 2 Department of Haematology, Royal Brisbane and Women’s Hospital, Butterfield St, Herston, QLD 4006 Australia; 3 School of Medicine, The University of Queensland, Brisbane, QLD Australia

**Keywords:** Hemophagocytic lymphohistiocytosis, Pembrolizumab, Melanoma, Immunotherapy, Case report

## Abstract

**Background:**

Hemophagocytic lymphohistiocytosis is rare and does not have any distinct clinical features or laboratory abnormalities, whereby a high index of suspicion is required for diagnosis. Hemophagocytic lymphohistiocytosis can be divided into primary/genetic or secondary causes that can be immunotherapy-induced. With the increasing use of immune checkpoint inhibitors for treatment of multiple solid tumor malignancies, the incidence of rare complications such as hemophagocytic lymphohistiocytosis can be expected to rise.

**Case presentation:**

We describe a fatal case of immunosuppression-refractory pembrolizumab-induced hemophagocytic lymphohistiocytosis complicated by secondary disseminated fungal infection. A 75-year-old white Australian man with resected stage IIC melanoma who received four cycles of adjuvant pembrolizumab presented 11 weeks after commencement with febrile neutropenia, elevated C-reactive protein, and nonspecific symptoms. He developed progressive transaminitis, acute kidney injury, pancytopenia, hyperferritinemia, and hypofibrinogenemia. In addition, a bone marrow aspiration and trephine biopsy demonstrated hemophagocytosis. Treatment was commenced with high-dose corticosteroids and mycophenolate mofetil initially for suspected immune-related side effects, followed by tocilizumab and anakinra once a diagnosis of hemophagocytic lymphohistiocytosis was made. Unfortunately, his disease was refractory to treatment with development of multiorgan failure secondary to hemophagocytic lymphohistiocytosis, complicated by disseminated candidemia from prolonged immunosuppression, leading to death 26 days after admission.

**Conclusion:**

This case underscores the diagnostic complexities of hemophagocytic lymphohistiocytosis, the importance of a multidisciplinary approach to management, and the fatal side effects of extended immunosuppression. This also highlights the importance of the discussion of risks versus benefits of treatment, particularly in the adjuvant setting, emphasizing the rare but real risk of fatal toxicities. Prolonged immunosuppression can lead to severe complications, and judicious use of corticosteroids with intensive prophylaxis is crucial. Further research into the mechanism of checkpoint inhibitor-induced toxicities is critical in the era of immunotherapy to allow personalized immunosuppressive and steroid-sparing strategies in complex cases.

## Background

Hemophagocytic lymphohistiocytosis (HLH) is a dysregulated systemic inflammatory state with persistent activation of natural killer (NK) cells, cytotoxic T lymphocytes, and macrophages, leading to elevation of serum proinflammatory cytokines causing immune-related multiorgan damage. HLH can be broadly divided into primary/familial due to a predisposing underlying genetic defect in immune function and secondary, which arises due to external triggers, most commonly infection, malignancy, or rheumatological disease (termed macrophage activation syndrome) [1]. HLH is rare, with sparse epidemiological data in adults, ranging from 1 in 2000 adult admissions in tertiary centers [2] to 1 in 800,000 cases per year [3].

Immune checkpoint inhibitors (ICI) have revolutionized the treatment paradigm of malignancies in recent years with increasing use across various tumor subtypes. Anti-programmed death protein 1 (PD-1), programmed cell death-ligand 1 (PD-L1), and cytotoxic T-lymphocyte associated antigen 4 (CTLA-4) ICIs act as negative regulators of T-cell immune function, resulting in increased activation of the immune system [4]. Immune-related adverse effects (IRAEs) are more common with anti-CTLA-4 compared with anti PD-1/PD-L1-directed agents. Severe (≥ grade 3) immune-related adverse effects have a prevalence of 14–20%, but fatal IRAEs are exceedingly rare (< 1%) [5, 6]. 

There have been increasing reports of ICI-induced HLH, and we describe a rare case of ICI-associated HLH in a patient receiving adjuvant pembrolizumab for a resected stage IIC melanoma.

## Case presentation

The patient was a 75-year-old white Australian man who had a resected stage IIC (pT4b Nx M0) BRAF V600E-mutant melanoma. Sentinel lymph node biopsy was not performed for staging as his disease was deemed high risk with a 57% sentinel node metastasis risk based on the Melanoma Institute of Australia risk calculator [7, 8]. He had an Eastern Cooperative Oncology Group (ECOG) performance status of 0 with no significant past medical history or medications. He was commenced on adjuvant PD-1 inhibitor pembrolizumab (200 mg every 21 days), completing four cycles with good tolerance. Restaging fluorodeoxyglucose (FDG)-positron emission tomography (PET) scan after the fourth cycle did not show any evidence of recurrent disease.

He was admitted to hospital 78 days (11 weeks) after commencement of pembrolizumab and 15 days post his fourth cycle for febrile neutropenia (neutrophils of 0.02 × 10^9^/L) with no localizing symptoms. C-reactive protein (CRP) was elevated at 298 mg/L with normal hemoglobin, platelets, liver, and renal function. Septic work-up, including chest X-ray, blood cultures, urine microscopy, and respiratory viral panel were unremarkable. Treatment was commenced with intravenous piperacillin-tazobactam, a single dose of intravenous gentamicin, and granulocyte colony-stimulating factor (G-CSF). Supportive measures with intravenous fluids and packed red blood cell transfusions were implemented throughout the admission.

**Fig. 1:**
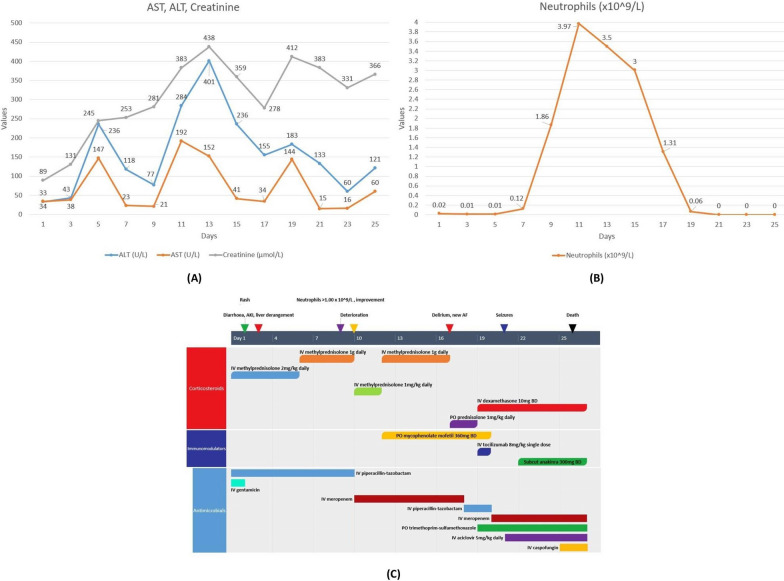
**A** Alanine transaminase, aspartate transaminase, and creatinine levels over time, demonstrating progressive liver and renal injury despite treatment with broad-spectrum antibiotics, methylprednisone, and mycophenolate. There was marginal improvement following administration of tocilizumab and dexamethasone on day 19, corresponding to a brief period of clinical improvement with fever resolution. **B** Neutrophil counts over time. Although a brief increment was seen after several days of granulocyte colony-stimulating factor, there was progressive decline from day 10 to a nadir of 0 × 10^9^/L, corresponding to the gradual deterioration in the patient’s clinical condition. **C** Timeline of treatments and key changes in the patient’s clinical condition. The colored arrows indicate key clinical changes in the patient’s condition corresponding to the day of admission. BD = twice a day, PO = per os/by mouth, IV = intravenous

From day 2–4, he developed a generalized erythematous papular rash, grade 2 diarrhea (based on Common Terminology Criteria for Adverse Events [CTCAE] version 5), grade 3 acute kidney injury (AKI), grade 3 transaminitis (Fig. 1A), and elevated ferritin levels of 1610 µg/L. In addition, he remained febrile above 39 °C. Computed tomography (CT) of the chest, abdomen, and pelvis was unremarkable with no organomegaly. The patient remained persistently neutropenic despite daily G-CSF, and repeated blood cultures were negative throughout. Further infectious work-up was negative for hepatitis B and C, human immunodeficiency virus (HIV), cytomegalovirus (CMV), and Epstein–Barr virus (EBV). Intravenous vancomycin was added to broaden antimicrobial coverage, and intravenous methylprednisolone 2 mg/kg/day was commenced for presumed IRAE, which was later increased to 1 g daily owing to minimal improvement.

Patient demonstrated clinical and hematological improvement from day 5 to day 9, with resolution of neutropenia; therefore, antibiotics were ceased. Bone marrow aspiration and trephine (BMAT) biopsy was performed, which demonstrated adequate granulopoiesis and no hemophagocytosis, noting that biopsy was performed on the day of neutrophil recovery (Fig. 1B).

From day 10 onward, his clinical condition took a downturn with fever recurrence and fluid overload, along with deterioration in renal and liver function. Transthoracic echocardiogram was unremarkable. Intravenous methylprednisolone dose was reduced to 1 mg/kg/day to prevent further exacerbation of edema, and mycophenolate mofetil (MMF) 360 mg twice a day was added. Antibiotics were restarted with intravenous meropenem owing to concerns for possible penicillin-induced acute interstitial nephritis contributing to his acute kidney injury (AKI). Oral trimethoprim-sulfamethoxazole was also introduced for *Pneumocystis jirovecii* prophylaxis. After minimal improvements in 2 days, intravenous methylprednisolone was increased to 1 g daily, later complicated by hematochezia and melena, treated conservatively with intravenous pantoprazole.

**Fig. 2:**
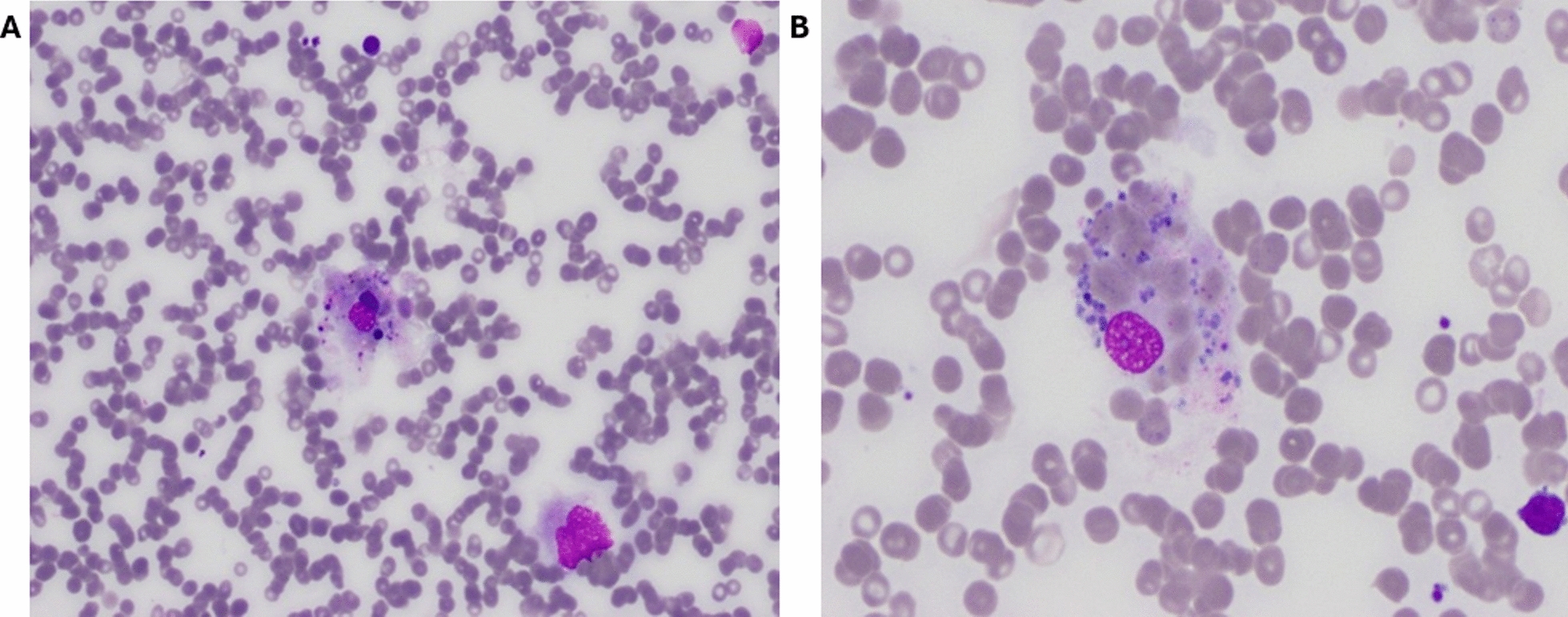
Aspirate slides from the second bone marrow biopsy with hematoxylin and eosin staining demonstrating a hypocellular marrow with prominent hemophagocytosis. Magnification 20X (**A**) and 40X (**B**)

On day 17, the patient rapidly deteriorated with new neurological dysfunction with fluctuating cognition, new atrial fibrillation, ongoing fevers, pancytopenia (nadir neutrophil count of 0.00 × 10^9^/L, platelets of 36 × 10^9^/L, and hemoglobin of 92 g/L on day 20), elevated ferritin of 7460 µg/L, and low fibrinogen of 1.2 g/L. Clinical suspicion of HLH was made, and a repeat BMAT biopsy on day 19 was carried out, indicating the presence of histiocytes with erythrophagocytosis and a mildly hypocellular marrow with reduced granulopoiesis (Fig. 2). At this point, the patient met the HLH-2004 diagnostic criteria with fever, pancytopenia, elevated ferritin, hypofibrinogenemia, and hemophagocytosis on bone marrow. The calculated H-score was 261, corresponding to a 99% probability for HLH. A single dose of tocilizumab (interleukin-6 receptor monoclonal antibody) was administered on day 19, steroids changed from methylprednisone to intravenous dexamethasone 10 mg twice daily, and MMF was ceased in the context of pancytopenia.

The patient demonstrated a brief period of clinical improvement with fever resolution and decreased ferritin level to 1600 µg/L but deteriorated again on day 21 with new seizures. This was managed with intravenous levetiracetam, and intravenous acyclovir was started empirically for possible herpes simplex virus (HSV) encephalitis given his prolonged immunosuppression. Magnetic resonance imaging (MRI) of the brain did not demonstrate features of encephalitis. Lumbar puncture was negative for viral, bacterial, cryptococcal, and autoimmune panels. Anakinra 300 mg subcutaneously twice daily was commenced on day 22 for treatment-refractory HLH; however, the patient continued to progress to septic shock with oliguric renal failure and decreased consciousness. He was admitted to the intensive care unit for intubation, noradrenaline infusion, and continuous renal replacement therapy. Two sets of blood cultures were positive for *Candida tropicalis*, and computed tomography (CT) of the abdomen showed multiple hypodensities throughout the liver and spleen suggestive of abscesses in the setting of disseminated candidemia. Patient died on day 26 of admission. A timeline of treatments and key changes in the patient’s clinical condition is summarized in Fig. 1C. 

## Discussion and conclusion

This case was complex, with multiple diagnostic conundrums and a fluctuating clinical course. The patient ultimately succumbed to multiorgan failure from HLH complicated by secondary disseminated candidemia due to prolonged immunosuppression.

Of adult-onset HLH cases, 70–80% are malignancy-associated, more commonly associated with hematological cancers [9, 10]. Proposed hypotheses for its mechanisms include a hyperinflammatory state triggered by malignancy causing excessive proinflammatory cytokines release, inherited immune disorders predisposing patients to both cancer and HLH, chemotherapy-induced immune dysregulation, and immunosuppression causing secondary infections acting as a trigger for HLH [11].

With the increasingly widespread use of ICIs in solid organ malignancies, there have been a few reports of ICI-induced HLH in literature. Owing to the rarity of this condition, the exact mechanism of ICI-induced HLH is poorly understood. A case series of three patients with ICI-associated HLH demonstrated elevated levels of circulating proinflammatory cytokines (for example, IL-6, IL-18, and CXCL9) and low levels of natural cytokine inhibitors (for example, IL-1RA and IL-10), suggesting a possible shared pathogenesis between ICI-associated HLH and other secondary causes [12]. One study found a negative correlation between PD-1 expression on tumor-associated macrophages and its phagocytic potency in mouse and human models of cancer [13]. This is consistent with the mechanism of action of ICI, which creates a hyperinflammatory state that characterizes HLH.

Our knowledge on the epidemiology of ICI-associated HLH predominantly arises from case series or individual case reports. ICI-associated HLH is an extremely rare IRAE, with a reported incidence between 0.03% and 0.4% [14] and a 30-day mortality rate of up to 44% [2]. Majority of the cases received an anti-PD-1 or PD-L1 agent for melanoma followed by lung cancer [14, 15], likely owing to earlier use of ICI in these two tumor streams. Time from ICI initiation and onset of HLH varies, ranging from 5 days to 1 year. In previously reported cases, almost all patients received corticosteroids and a combination of chemotherapy (etoposide), immunosuppression (mycophenolate, tacrolimus, cyclosporine), intravenous immunoglobulin (IVIG), tocilizumab, and anakinra [9, 15–17]. Specific risk factors for ICI-associated HLH are not known. A single case involving a patient with metastatic breast cancer who developed pembrolizumab-induced HLH was found to have an underlying PRF1A91V germline polymorphism [18], a gene implicated in cases of primary/familial HLH [1]. These genetic aberrations are not routinely tested in our current practice, and whether other cases of ICI-associated HLH have a similar genetic predisposition is not known.

Presentation of HLH is heterogeneous, and diagnosis is often challenging with varied clinical and laboratory abnormalities. A diagnosis is often made using the HLH-2004 criteria when one of two criteria are met: molecular diagnosis consistent with HLH and/or meeting five out of eight diagnostic criteria (fever, splenomegaly, cytopenias of two or more lineages, hypertriglyceridemia and/or hypofibrinogenemia, hemophagocytosis in the bone marrow or spleen or lymph nodes, low or absent NK-cell activity, ferritin ≥ 500 µg/L, soluble CD25 ≥ 2400 U/ml) [19]. The H-score is a web-based calculator based on graded clinical and laboratory parameters, which is a validated diagnostic tool for adults [20]. More commonly, these criteria are only partially met, and HLH is diagnosed and treated on the basis of a high index of clinical suspicion. Pathologic alterations due to HLH may also often take days to weeks to manifest [11], evidenced by the initial negative BMAT biopsy for HLH in our case. There are no specific features distinguishing ICI-induced HLH from other secondary causes.

There is increasing recognition of HLH as a complication of novel targeted therapies in addition to immune checkpoint inhibitors. For example, HLH has been documented in patients who have undergone chimeric antigen receptor T-cell (CAR-T) therapy in hematological malignancies. Guidelines based on prospective trials in adults for the management of these treatment-associated HLH currently do not exist. International oncology management guidelines such as the American Society of Clinical Oncology (ASCO) and the European Society for Medical Oncology (ESMO) acknowledged the rarity of hematological IRAEs, with general recommendations of corticosteroids, withholding ICIs, early hematologist involvement, and second-line immunosuppressants (for example, IVIG, rituximab, MMF, cyclosporine) for refractory cases [21, 22]. ESMO guidelines recommended consideration for anti-IL-6R therapy (tocilizumab) for HLH [22].

Known HLH management protocols such as HLH-94 and HLH-2004 were developed for children and, therefore, were not validated in the adult population. Both protocols utilize corticosteroids, cyclosporine A, intrathecal therapy, and etoposide, followed by bone marrow transplantation, with differences in timing and sequence of therapies [10]. The HLH group has published expert opinions on the management of adult ICI-associated HLH with recommendations for treatment with corticosteroids and anti-IL-6R therapy (tocilizumab) in patients with suspected HLH, followed by consideration of etoposide if there is insufficient response after 48 hours [10, 23]. Tocilizumab has been reported to be effective in a published case series of three patients with metastatic melanoma complicated by ICI-associated HLH, showing rapid clinical improvement and normalization of laboratory markers and proinflammatory cytokine levels [12]. IVIG, anakinra, and corticosteroids have been used in practice and shown benefit [24], whilst the benefit of mycophenolate mofetil and tacrolimus has only been reported in case reports of HLH in rheumatological conditions [25, 26]. Given the high mortality rates seen in ICI-associated HLH and our increasing use of immune-targeted therapies, there is a need to establish guidelines tailored toward these patients to improve outcomes. In our case, the most likely cause was pembrolizumab, as secondary causes for HLH such as active malignancy and infection was ruled out by a normal FDG-PET scan and repeatedly negative infective screens. G-CSF has previously been reported to cause secondary HLH [27]. However, the patient’s clinical symptoms and neutropenia preceded G-CSF administration, which suggested the process of HLH commenced prior to G-CSF administration. With prolonged immunosuppression, this led to disseminated candidemia, which was ultimately fatal in our case.

This case highlights the importance of thorough patient–clinician discussions regarding risks and benefits of treatment, particularly in the adjuvant setting. With widespread adoption of ICI, it is likely that cases of rare and fatal IRAEs such as HLH will continue to rise. ICI-associated HLH requires a high index of suspicion, and a multidisciplinary approach is crucial owing to the lack of clear management guidelines. As evidenced by our case, prolonged high-dose immunosuppression can lead to fatal consequences. Adequate infection prophylaxis should be undertaken with the aim to down-titrate to the lowest possible corticosteroid dose along with the use of steroid-sparing agents. Further research into the mechanism of checkpoint inhibitor-induced toxicities is critical in the era of immunotherapy to allow personalized immunosuppressive and steroid-sparing strategies in complex cases.

## Data Availability

The datasets used and/or analyzed during the current study are available from the corresponding author on reasonable request.

## Abbreviations

HLH: Hemophagocytic lymphohistiocytosis
ICI: Immune checkpoint inhibitor
NK: Natural killer
PD-1: Anti-programmed death protein 1
PD-L1: Programmed cell death-ligand 1
CTLA-4: Cytotoxic T-lymphocyte associated antigen 4
IRAE: Immune-related adverse effect
ECOG: Eastern Cooperative Oncology Group
FDG-PET: Fluorodeoxyglucose-positron emission tomography
CRP: C-reactive protein
G-CSF: Granulocyte colony-stimulating factor
IV: Intravenous
CTCAE: Common terminology criteria for adverse events
AKI: Acute kidney injury
CT: Computed tomography
BMAT: Bone marrow aspiration and trephine
MMF: Mycophenolate mofetil
HSV: Herpes simplex virus
CAR-T: Chimeric antigen receptor T cell
